# Melatonin Supplementation Relieves Fluoride-Induced Bone Injury via Ion Homeostasis Disorder and PINK1/Parkin-Mediated Mitophagy

**DOI:** 10.3390/foods14244173

**Published:** 2025-12-05

**Authors:** Cuicui Zhuang, Jinhui Zhao, Xinying Zhang, Mingyue Guo, Yiguang Lu, Ting Pei, Yangfei Zhao, Chen Liang, Jianhai Zhang

**Affiliations:** 1 College of Veterinary Medicine, Shanxi Agricultural University, Taigu 030801, China; 19335945779@163.com (J.Z.); zxywencydd@163.com (X.Z.); y741250879@163.com (M.G.); 15635430550@163.com (Y.L.); peiting202103@163.com (T.P.); zyf91sky@163.com (Y.Z.); 2 College of Animal Science, Shanxi Agricultural University, Taigu 030801, China; cekiv@163.com

**Keywords:** skeletal fluorosis, melatonin, ion homeostasis, PINK1/Parkin pathway, mitophagy

## Abstract

Long-term excessive fluoride intake from food causes skeletal fluorosis, which manifests as bone sclerosis, deformation, joint dysfunction, and even disability. Mitophagy and ion homeostasis regulate bone function. This study investigated the role of melatonin (MLT) in mitigating this condition, given its known involvement in bone remodeling and the fact that fluoride impairs its synthesis in the pineal gland. Firstly, network pharmacology and molecular docking identified mitophagy as MLT’s key pathway against sodium fluoride (NaF)-induced osteosclerosis. Subsequently, a 400 mg/kg/day body weight NaF exposure model in chicken model with 25 mg/kg/day body weight MLT intervention were established in the current study. Fluoride exposure caused the disturbance of ion homeostasis, and the impairment of mitochondria and activation of PTEN-induced putative kinase1 (PINK1)/E3 ubiquitin ligase Park2 (Parkin)-mediated mitophagy in the bone. Importantly, these deleterious effects were significantly restored by MLT supplementation. In conclusion, NaF causes bone injury via ion homeostasis disruption, osteoblast mitochondrial damage, leading to excessive mitophagy. MLT inhibits fluoride-induced mitophagy through the calcium ion flow-mediated PINK1/Parkin pathway, mitigating bone damage. This study can not only ensure the safety of animal-derived food but also provide a theoretical basis for the prevention and treatment of fluorosis in humans and animals.

## 1. Introduction

Fluorine is a highly reactive halogen. In biological systems and environmental media, it primarily exists as fluoride ions [1]. For humans and livestock, dietary intake is a major route of fluoride exposure. Common sources include seafood (especially products derived from skeletal tissues), tea leaves, animal bone supplements, crops from fluoride-rich regions. Food-processing additives and contaminated drinking water are also significant sources. Ingestion of fluoride-contaminated food poses serious health risks. It also compromises the safety and quality of animal-derived food products, which is a key concern for food systems [2]. Despite global regulations having set intake limits, fluorosis remains a widespread public health issue. It affects approximately 180 million people worldwide, with the highest rates in Asia and Africa [3]. Over 90% of ingested fluoride accumulates in mineralized tissues, like bones and teeth. This leads to two primary conditions: dental fluorosis and skeletal fluorosis. Chronic exposure to fluoride concentrations >1.5 mg/L (e.g., from drinking water or fortified foods) induces dental fluorosis. When prolonged daily intake exceeding 6 mg, it can progress to severe, disabling skeletal fluorosis [4]. The research on fluoride’s skeletal effects began in the mid-20th century [5]. However, fluorosis continues to threaten bone health in both human and animal populations. Critically, fluoride bioaccumulated in livestock can enter the human food chain. This occurs through the consumption of products like poultry bones or bone-in meat, thereby exacerbating dietary exposure. Despite this persistent threat, the molecular mechanisms of skeletal fluorosis are still not fully understood. Furthermore, no targeted therapies are available for treating this condition in clinical or veterinary practice. Addressing these research gaps is therefore essential. It is a crucial step toward protecting animal health and ensuring the safety of the global food supply.

Autophagy is the primary intracellular degradation machinery. It transports organelles, proteins, and macromolecule to lysosomes for degradation. This process sustains cellular renewal and maintains intracellular homeostasis [6]. There are two main subtypes: non-selective autophagy, typically activated by nutrient starvation, and selective autophagy, which specifically targets damaged proteins or dysfunctional organelles [7]. Mitophagy is a specialized form of selective autophagy. It mediates the clearance of impaired mitochondria via receptor-dependent mechanisms. Under normal conditions, it acts as a physiological housekeeping process. Its activity increases under pathological states or specific stimuli to eliminate excessive or damaged mitochondria, thereby preserving mitochondrial quality [8]. Growing evidence indicates that dysregulated autophagy and mitophagy disrupt bone metabolism balance and contribute to bone metabolic disorders [9,10]. The most well-characterized mitophagy pathway in mammalian cells involves PTEN-induced putative kinase 1 (PINK1) and the E3 ubiquitin ligase Parkin [11]. Upon activation, Parkin ubiquitinates mitochondrial proteins. This recruits the receptor protein SQSTM1/p62, which then binds to microtubule-associated protein 1 light chain 3 (LC3). This binding facilitates the engulfment of damaged mitochondria into autophagosomes. Additionally, the outer mitochondrial membrane proteins BNIP3 and NIX can trigger mitophagy under ischemic or hypoxic conditions. BNIP3, for instance, releases Beclin-1 via its BH3 domain and directly binds LC3 via its N-terminal LIR motif [12]. Previous studies have demonstrated that fluoride exposure induces mitophagy and apoptosis in MLO-Y4 osteoblasts and mouse tibiae by promoting excessive reactive oxygen species (ROS) production [13,14]. However, the relationship between mitophagy and skeletal fluorosis in chickens remains unclear. Elucidating this connection represents a critical knowledge gap in current research.

Melatonin (MLT, N-acetyl-5-methoxytryptamine) is a hormone. It is primarily synthesized and secreted by the pineal gland at night. MLT is present in all humans and animals but distributes unevenly within cells [15]. Beyond well-known role in regulating circadian rhythms and sleep, MLT is a key modulator. It influences anti-inflammatory, antioxidant, and anti-autophagic processes [16,17]. Notably, high MLT concentrations accumulate in mitochondria. This enhances cellular resistance to oxidative stress and apoptosis. This property is critical for maintaining organelle function in metabolically active tissues like bone. Endogenous MLT levels follow circadian cycles and typically decline with age [18,19]. Exogenous MLT is widely used as a dietary supplement. The U.S. Food and Drug Administration generally recognizes it as safe [20]. It is readily absorbed across cell membranes and distributed throughout the body. This raises serum MLT levels, which can alleviate various pathological conditions [21,22]. A critical link to food safety exists. Fluoride often accumulates in the body via contaminated food or drinking water. Studies show that it also accumulates in the pineal gland, thereby impairing MLT synthesis [23]. Importantly, MLT regulates both bone formation and resorption. It has shown beneficial effects on bone disorders like osteoarthritis and rheumatoid arthritis, primarily by inhibiting autophagy [24,25]. However, its role in skeletal fluorosis remains uncharacterized. This condition is directly linked to dietary fluoride exposure. Furthermore, it is still unknown whether exogenous MLT can mitigate skeletal fluorosis by regulating autophagic pathways. This requires further investigation.

This study used a high-dose pharmacological model to define sodium fluoride (NaF) toxicity and MLT protective mechanisms. This approach, while not reflective of typical environmental exposure, is highly effective for uncovering clear biochemical and pathological pathways. We aimed to clarify the mechanism of fluoride-induced bone damage and to elucidate how MLT counteracts this harm. The findings are expected to provide critical insights for enhancing the safety of poultry-derived foods and for developing novel, nutrition-integrated strategies to manage fluorosis in both veterinary and human medicine.

## 2. Materials and Methods

### 2.1. Network Pharmacology

To investigate the potential protective mechanisms of melatonin (MLT) against sodium fluoride (NaF)-induced skeletal fluorosis, we applied a network pharmacology approach. The workflow consisted of the following steps. First, target genes associated with the disease and intervention were retrieved from specialized databases. The keywords “melatonin”, “sodium fluoride”, and “osteosclerosis” were used to search the Comparative Toxicogenomics Database (CTD; https://ctdbase.org/) [26], while “sodium fluoride” and “osteosclerosis” were employed to query the GeneCards Database (https://www.genecards.org/)—a comprehensive repository of human gene information [26]. Concurrently, potential molecular targets of MLT were predicted via SwissTargetPrediction (http://swisstargetprediction.ch), a tool optimized for predicting small-molecule binding partners in selected species [27]. Next, the gene sets obtained from the above databases were intersected to identify candidate target genes mediating MLT’s protective effects against NaF-induced skeletal fluorosis. These candidate genes were then subjected to Gene Ontology (GO) functional annotation and Kyoto Encyclopedia of Genes and Genomes (KEGG) pathway enrichment analysis using Metascape (http://metascape.org/gp/index.html; accessed on 28 March 2025), a platform for high-throughput functional enrichment of gene lists [26]. To further prioritize key targets, Metascape was combined with Cytoscape software (version 3.9.1, Cytoscape Consortium, San Diego, CA, USA) and its Cluego plugin for network construction and module analysis [26]. Finally, molecular docking was performed to validate the binding affinity between MLT and the key target proteins using CB-Dock2 (https://cadd.labshare.cn/cb-dock2/php/index.php; accessed on 30 March 2025)—an advanced tool for blind protein-ligand docking that integrates cavity detection and homologous template fitting [28]. A binding energy threshold of <−5 kcal/mol was applied to define favorable binding interactions, as this value is widely recognized to indicate robust ligand-target binding activity [29].

### 2.2. Animal Treatment

This study was approved by the Animal Care and Use Ethics Committee of Shanxi Agricultural University, China (Protocol Number: SXAU-EAW-2022P.AD.006007238), and the approval certificate is available upon reasonable request. All experimental procedures involving animals were conducted in strict compliance with this approved protocol. One-day-old male Hylan Brown chickens (*n* = 32) were purchased from Shanxi Kangmuyuan Animal Husbandry Co., Ltd. (Jinzhong, China) and subjected to a 3-week acclimation period prior to the experiment. During acclimation and the entire experimental period, chickens were housed under standardized conditions: ad libitum access to drinking water and a commercial formulated feed (purchased from Jinzhong Shiyang Feed Co., Ltd., Jinzhong, China) with a fluoride concentration of <20 mg/kg (see Table S1 for details) and nutrient composition as specified in Table S2. Routine vaccination and standardized feeding management were implemented throughout. Housing environmental parameters were controlled as follows: an initial temperature of 34 °C for the first 2 days, which was gradually reduced by 3 °C per week until reaching 21 °C (maintained until the experiment ended); and a light regime of 24 h/day for the first 2 days, gradually reduced to 18 h/day by day 21 (sustained thereafter). After three weeks of adaptability, 32 one-day old male Hylan Brown chickens purchased from Shanxi Kangmuyuan Animal Husbandry Co., Ltd. (Jinzhong, China) were randomly divided into 4 groups: control, NaF (400 mg/kg/day NaF, orally), MLT (25 mg/kg/day melatonin (MLT), orally), and NaF+MLT (400 mg/kg/day NaF, orally; and 25 mg/kg/day MLT, orally) for 32 weeks. NaF powder (purity ≥ 99%; Sigma-Aldrich, Shanghai, China; cat # 201154) was dried at 120 °C for 12 h, then dissolved in distilled water and prepared as a NaF solution, which is then mixed with formulated feed. A trace amount of melatonin (purity ≥ 99%; Shenggong Bioengineering (Shanghai, China) Co., Ltd.; cat # A600605) dissolved in 95% ethanol (Aladdin, Riverside, CA, USA; cat # A112717) was mixed with water and then mixed with formulated feed. At the end of the 32-week treatment, all chickens were humanely euthanized. Serum samples were collected, centrifuged to separate serum, and stored at −80 °C for subsequent analyses. Tibias were harvested immediately: portions were stored in an ultracold freezer, while others were fixed in 10% neutral buffered formalin (Aladdin; cat # F301880) for micro-computed tomography (micro-CT) scanning. Additionally, tibial epiphyses were fixed in either 10% neutral buffered formalin or 2.5% glutaraldehyde (Aladdin; cat # G1373507) for subsequent histological examination.

### 2.3. Analysis of Bone Metabolism

Serum was used to detect activities of alkaline phosphatase (ALP; Nanjing Jiancheng Bioengineering Institute, Nanjing, China; cat # A059-2-2) and tartrate-resistant acid phosphatase (TRAP; Nanjing Jiancheng Bioengineering Institute; cat # A015-5-1), and osteocalcin (OCN; Nanjing Jiancheng Bioengineering Institute; cat # H152-1-2) concentration through ALP, TRAP and OCN assay kits according to the manufacturers’ instructions.

### 2.4. Histopathological Assessment of Tibiae Epiphyses

Histopathological assessments of liver tissue damage were performed using hematoxylin-eosin (H&E; Aladdin; cat # E489517) and Masson’s (Aladdin; cat # M774209) trichrome staining. For tibial tissue analysis, fixed samples underwent decalcification, followed by dehydration through a graded ethanol series, clearing in xylene, and embedding in paraffin. Paraffin-embedded tissues were sectioned at 5 µm thickness and subjected to staining protocols: H&E staining for general histological observation, or Masson’s trichrome staining (using Weigert’s hematoxylin, Biebrich scarlet-acid fuchsin solution, and Aniline blue) to evaluate collagen deposition. Stained sections were visualized and imaged under a light microscope.

### 2.5. Radiological Evaluation of Tibias

Following fixation, tibial samples were scanned using a high-resolution micro-computed tomography (micro-CT) system (SkyScan 1276; Bruker Micro-CT, Kontich, Belgium) under the following parameters: 55 kV tube voltage, 200 μA tube current, a 0.25 mm aluminum–copper (Al-Cu) filter, and an isotropic voxel resolution of 6 μm. A 360° rotational scan was performed with a step size of 0.3° to ensure comprehensive tissue coverage. Raw scan data were reconstructed into visualizable images using NRecon v.1.6.3 software (Bruker Micro-CT), with post-processing parameters set as follows: ring artifact correction = 5, flat field correction = 3, and beam hardening correction = 30%. Reconstructed images were visualized and evaluated across three anatomical planes (transverse, coronal, and sagittal) to assess overall bone structure. Quantitative analysis of newly formed bone tissue was conducted using CTAn v.1.12 software (Bruker Micro-CT), with 3D bone structure models generated via CTVol v.2.2.1 software (Bruker Micro-CT) to facilitate structural assessment. The following bone microarchitectural parameters were measured: bone volume fraction (BV/TV), trabecular number (Tb.N), trabecular connectivity density (Conn.Dn), trabecular separation (Tb.Sp), trabecular thickness (Tb.Th), and tissue mineral density (TMD).

### 2.6. Ultrastructure Analysis of Tibiae Epiphyses

Following initial fixation, tibial epiphyseal samples were post-fixed in osmic acid to preserve ultrastructural details, and then dehydrated through a graded ethanol series. Subsequent infiltration was performed using two gradient solutions: a 1:1 mixture of propylene oxide and epoxy resin, followed by a 1:2 mixture of the same reagents. Samples were then embedded in pure epoxy resin and polymerized in ovens at sequential temperatures: 37 °C, 45 °C, and 60 °C, with each temperature stage maintained for sufficient curing time. Polymerized resin blocks were sectioned into 50 nm ultrathin sections using an ultramicrotome. Sections were stained with uranyl acetate (for nucleic acid and membrane enhancement) followed by lead citrate (for general ultrastructural contrast)—a standard double-staining protocol for transmission electron microscopy (TEM) analysis. Stained ultrathin sections were examined and imaged using a TEM to visualize subcellular structures.

### 2.7. Detection of 26 Elements

Tibial samples from chickens were first ashed in a muffle furnace. The ashed residues were then subjected to acid digestion and subsequent elemental analysis. For multi-element quantification, a modified inductively coupled plasma mass spectrometry (ICP-MS) method was used [30], with slight adaptations to optimize for bone tissue matrices. Briefly, 100 mg of ashed tibial tissue was homogenized and digested with 10 mL of 65% nitric acid (HNO_3_; Aladdin; cat # N433819) and 4 mL of 30% hydrogen peroxide (H_2_O_2_; Aladdin; cat # H433859) in a thermal digester, following the manufacturer’s recommended digestion protocol. After complete digestion, the mixture was diluted to a final volume of 10 mL with deionized water. This digested solution was then analyzed via ICP-MS to determine the concentrations of 24 elements: Ag, Al, As, Ba, Cd, Co, Cr, Cu, Fe, K, Li, Mg, Mn, Mo, Ni, P, Pb, Sb, Se, Si, Sn, Ti, V, and Zn. Separately, the fluoride (F^−^) concentration in the digested tibial solution was measured using a fluoride ion-selective electrode (ISE) method [31], while calcium (Ca) concentration was quantified with a commercial Ca assay kit, strictly adhering to the kit manufacturer’s instructions.

### 2.8. qRT-PCR Analysis

Total RNA was extracted from tibial tissues using TRIzol reagent (TransGen Biotech Co., Ltd., Beijing, China; cat # ET111-01-V2), following the manufacturer’s standard protocol to ensure high RNA purity and integrity. For complementary DNA (cDNA) synthesis, 1 μg of total RNA was reverse-transcribed using a commercial reverse transcription kit (TransGen Biotech Co., Ltd.; cat # AU341-02), with reactions conducted under conditions optimized to minimize non-specific amplification. Quantitative real-time polymerase chain reaction (qPCR) was performed on an MX3000Pro qRT-PCR system (Agilent Technologies, Santa Clara, CA, USA) using SYBR Green PCR Master Mix—a reagent choice ensuring sensitive and specific detection of target gene transcripts (TransGen Biotech Co., Ltd.; cat # AQ132-11). Glyceraldehyde-3-phosphate dehydrogenase (GAPDH; Proteintech, Wuhan, China; cat # 60004-1-Ig) was used as the housekeeping gene to normalize mRNA expression levels, accounting for potential variations in RNA input and reverse transcription efficiency. Primers for target genes and GAPDH were designed based on chicken gene sequences retrieved from the National Center for Biotechnology Information (NCBI) database (see Table S3 for primer sequences and specifications). Relative mRNA expression levels of target genes were calculated using the 2^−ΔΔCt^ method, a widely accepted approach for quantifying gene expression changes in qPCR experiments.

### 2.9. Western Blot Analysis

Protein extraction and Western blot analysis were performed on tibial epiphyseal tissues as follows: Tissues were lysed in radioimmunoprecipitation assay (RIPA; Beyotime, Shanghai, China; cat # P0038) buffer supplemented with 1% phenylmethylsulfonyl fluoride (PMSF; purity ≥ 98.5%; Beyotime; cat # ST2573) to inhibit proteolysis. Protein concentration was quantified using a bicinchoninic acid (BCA) Protein Assay Kit (Beyotime; cat # P0009), strictly following the manufacturer’s protocol. Equal amounts of protein (30–100 μg per sample) were separated by 10–12% sodium dodecyl sulfate-polyacrylamide gel electrophoresis (SDS-PAGE) and transferred onto nitrocellulose (NC) membranes (Beyotime; cat # FFN83) via the wet-transfer method. Membranes were blocked with 5% non-fat milk powder in Tris-buffered saline with Tween 20 (TBST; Aladdin; cat # T665952) at room temperature for 2 h to reduce non-specific binding, then incubated overnight at 4 °C with diluted primary antibodies targeting the proteins of interest. After three washes with TBST, membranes were incubated with horseradish peroxidase (HRP)-conjugated secondary antibodies (Proteintech; cat # SA00012) at room temperature for 2 h. Protein bands were visualized using an enhanced chemiluminescence (ECL; Beyotime; cat # P0018S) detection system. Band intensities were quantified using image analysis software, and relative protein expression levels were normalized to the housekeeping proteins β-actin (Proteintech; cat # 66009-1-Ig) or Lamin B (Proteintech; cat # 12987-1-AP) to account for loading variations.

### 2.10. Statistical Analyses

Statistical significance was analyzed using one-way analysis of variance via LSD test in SPSS 26.0, and expressed as mean  ±  standard error. Histograms generated with Graphpad Prism 8. Heatmap and correlation (available online: https://www.omicstudio.cn/tool/59) were analyzed using Spearman correlation analysis (*p* < 0.05). Asterisks (*) indicates statistical significance compared to the control group, * *p* < 0.05 and ** *p* < 0.01. Pound sign (#) indicates statistical significance compared to the NaF group, # *p* < 0.05 and ## *p* < 0.01.

## 3. Results

### 3.1. Primary Evaluation of the Chicken Model Exposed to Fluoride and MLT Intervention

**Figure 1:**
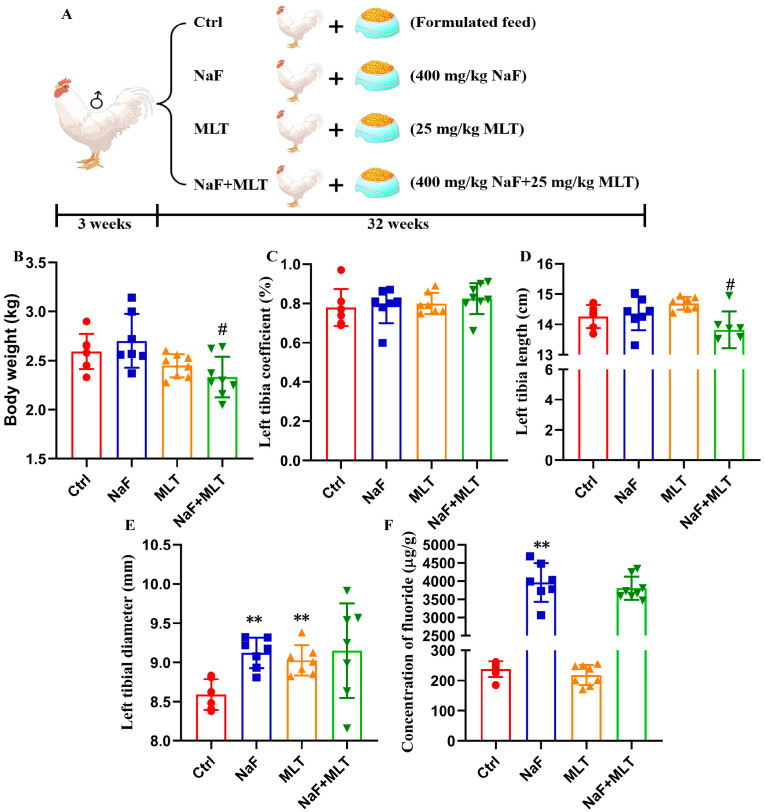
Fluoride exposure and melatonin intervention chicken model evaluation. (**A**) Schematic diagram of chicken model design and treatment. (**B**–**E**) Changes in body weight, left tibia coefficient, left tibial length, and left tibial diameter of chicken from the Ctrl, NaF, MLT, and NaF + MLT groups. (**F**) Fluoride concentrations in bone (tibia) of chicken. ** *p* < 0.01 indicates significant differences compared to the Ctrl group. # *p* < 0.01 indicates significant differences compared to the NaF group.

The experimental design consisted of recording and presenting the body weight of the chicken throughout the study, as depicted in Figure 1A,B. Body weight and left tibial length were significantly reduced (*p* < 0.05) after receiving treatment with MLT (Figure 1B,D). There was no statistically significant change in left tibia coefficient (Figure 1C). Left tibial diameter had significantly increased (*p* < 0.01) with fluoride treatment (Figure 1E), accompanied by a notable increase in fluoride concentration (*p* < 0.01) with exposure to fluoride (Figure 1F). These indicate that the fluoride treatment chicken model was established.

### 3.2. MLT Supplementation Restored the Fluoride-Induced Tibiae Damage

**Figure 2:**
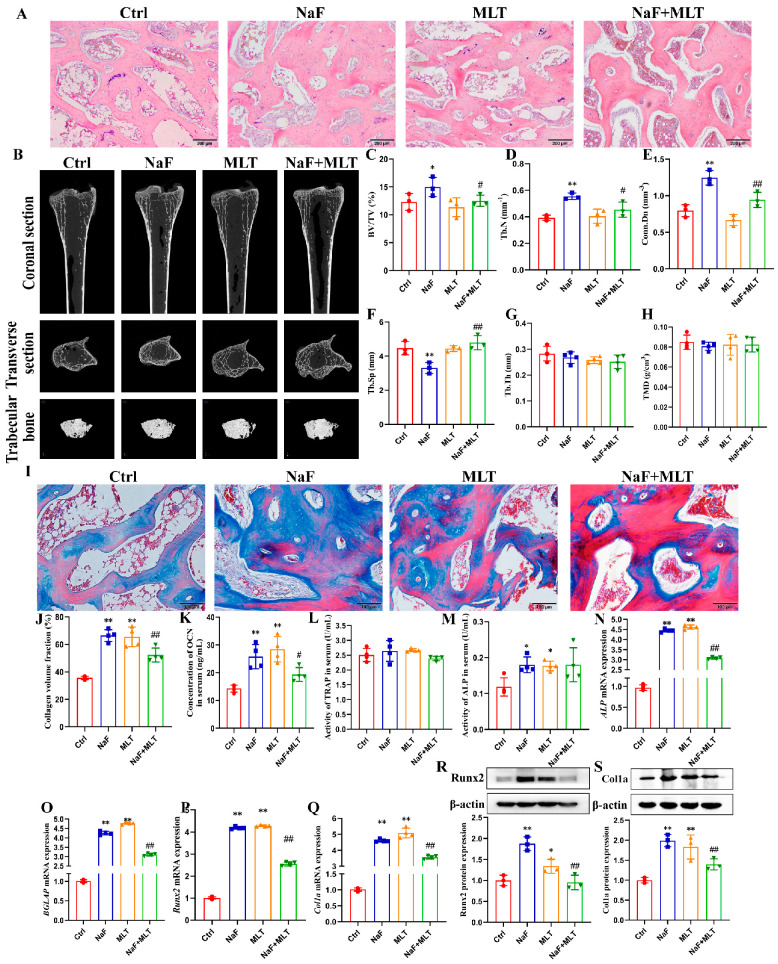
Fluoride induces bone morphology and function injury and melatonin intervention in chickens. (**A**) The representative morphological images of bone tissue in chicken (H.E staining). (**B**) Images from micro-CT scans, including coronal section, transverse section, and trabecular bone in chicken’s tibias. (**C**–**H**) The results of bone volume fraction (BV/TV), trabecular number (Tb.N), connectivity density (Conn.Dn), trabecular separation (Tb.Sp), trabecular thickness (Tb.Th), and tissue mineral density (TMD) from the analysis of micro-CT scans. (**I**) The representative morphological images of bone tissue in chicken (the representative morphological images of bone tissue in chicken (Masson staining). (**J**) Collagen volume fraction of bone tissue in chicken. (**K**–**M**) OCN concentration, and TRAP and ALP activities in serum of chickens. (**N**–**Q**) ALP, BGLAP, Runx2, and Col1a mRNA expressions in tibia of chicken. (**R**,**S**) Runx2 and Col1a protein expressions in tibia of chicken. * *p* < 0.05 and ** *p* < 0.01 indicate significant differences compared to the Ctrl group. ## *p* < 0.05 and # *p* < 0.01 indicates significant differences compared to the NaF group.

The histopathological changes in the tibiae epiphyses were observed by H.E staining and are shown in Figure 2A. The trabeculae of Ctrl group and NaF + MLT group were uniform and arranged neatly. In the NaF group, the pathologic manifestations of tibia epiphyseal tissue were bone marrow cavity narrowing, bone trabecular thickening, and disordered arrangement. The number of bone trabeculae increased in MLT group. Microscopic structure and morphology of the chicken tibia were scanned by micro-CT (Figure 2B). Bone volume fraction (BV/TV), trabecular number (Tb.N) and connectivity density (Conn.Dn) had significantly increased (*p* < 0.05; *p* < 0.01), accompanied by a notable decrease in trabecular separation (Tb.Sp; *p* < 0.01) with exposure to fluoride, which was relieved by MLT (*p* < 0.05; *p* < 0.01; Figure 2C–H). There was no statistically significant change in trabecular thickness (Tb.Th) and tissue mineral density (TMD; Figure 2G,H).

As shown in Figure 2I,J, Masson’s trichrome staining was used to detect collagen deposition in tibiae epiphyses. After fluoride exposure, collagen volume fraction was significantly increased (*p* < 0.01), which was relieved by MLT (*p* < 0.01). The administration of MLT significantly increased collagen volume fraction (*p* < 0.01). After fluoride or MLT treatment, OCN concentration and ALP activity were significantly increased (*p* < 0.05; *p* < 0.01), and MLT administration significantly reversed the effect of fluoride on OCN concentration (Figure 2K,L). There was no statistically significant change in TRAP activity (Figure 2M). After fluoride treatment, the mRNA expression levels of ALP, BGLAP, and Runx2, and ALP and Runx2 protein expressions significantly increased (*p* < 0.01), consistent with MLT supplement (*p* < 0.05; *p* < 0.01; Figure 2N–S). The administration of MLT significantly alleviated (*p* < 0.01) the high ALP, BGLAP and Runx2, and ALP and Runx2 protein expressions (Figure 2N–S).

### 3.3. Network Pharmacology Analysis of the Potential Fluoride-Induced Tibiae Damage of MLT

**Figure 3:**
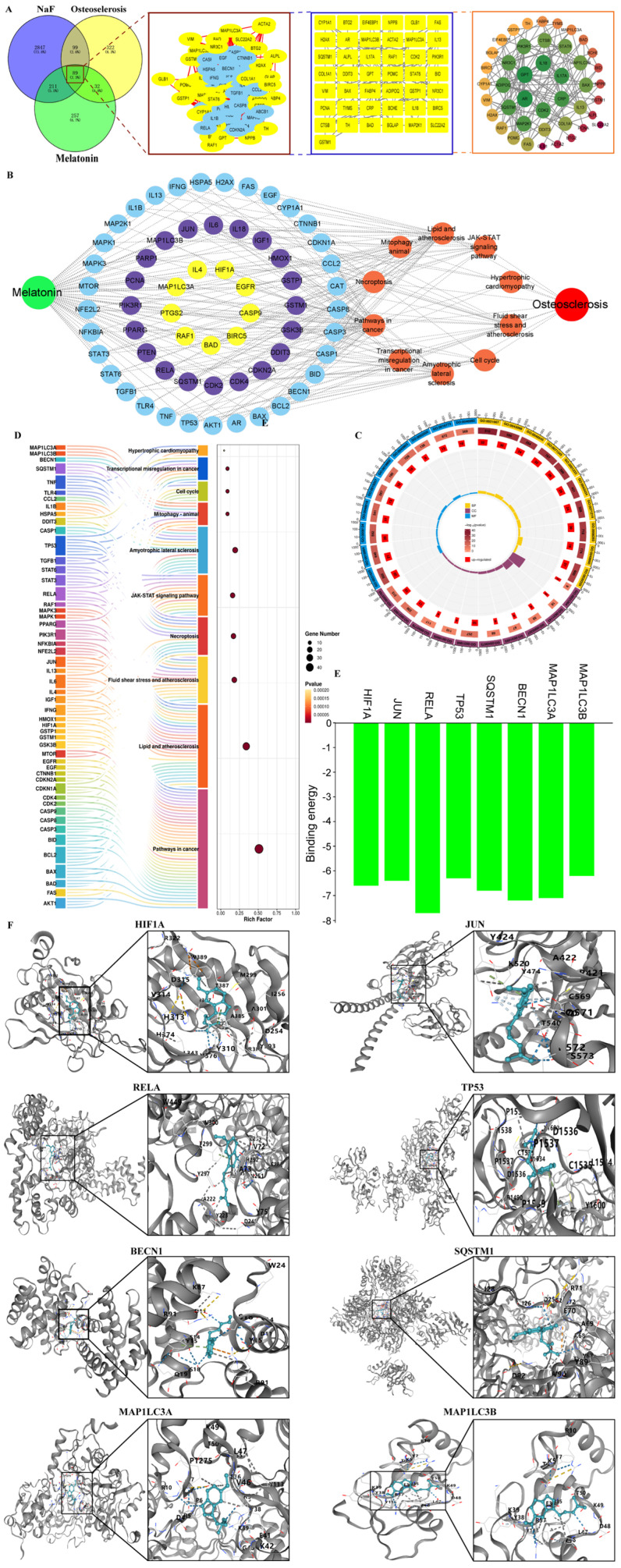
Network pharmacology analysis results. (**A**) Screening for key target genes of the protective of MLT on NaF-induced osteosclerosis. (**B**) The network map of MLT, target genes, pathways, and osteosclerosis in the top 10 pathways of KEGG enrichment after Metascape analysis. (**C**) GO enrichment circle diagram of MLT on NaF-induced osteosclerosis target genes. (**D**) KEGG enrichment results of MLT on NaF-induced osteosclerosis target genes. (**E**) The binding energies of MLT with HIF1A, JUN, RELA, TP53, BECN1, SQSTM, MAP1LC3A, or MAP1LC3B, respectively. The red line refers to the binding energy of −5 kcal/mol. (**F**) Molecular docking results of MLT and HIF1A, JUN, RELA, TP53, BECN1, SQSTM, MAP1LC3A, or MAP1LC3B, respectively.

Network pharmacology was employed to identify and analyze key target genes and biological characteristics associated with MLT. As shown in Figure 3A, 3246 NaF-related target genes, 598 MLT-related target genes, and 5542 osteosclerosis-related target genes were screened out, and a further intersection of these three datasets revealed 89 overlapping target genes. Gene ontology (GO) enrichment analysis revealed that the genes were primarily associated with response to nutrient levels, positive regulation of programmed cell death, death-inducing signaling complex, pseudopodium, ubiquitin protein ligase binding, protein domain specific binding, and so on (Figure 3C and Figure S1 and Table S4). In addition, KEGG enrichment analysis indicated that pathways in cancer, JAK-STAT signaling pathway, necroptosis, cell cycle, mitophagy—animals, and others played crucial roles in the protective effects of MLT against fluoride-induced osteosclerosis (Figure 3B,D, and Table S4). In addition, analysis revealed that mitophagy—animal pathway is involved in six genes (HIF1A, JUN, RELA, TP53, BECN1, SQSTM1, MAP1LC3A, and MAP1LC3B), and molecular docking results indicated that the HIF1A, JUN, RELA, TP53, BECN1, SQSTM1, MAP1LC3A, and MAP1LC3B proteins had multiple binding pockets that can stably bind to MLT, respectively (Figure 3F). MLT demonstrated the lowest binding energies with HIF1A (−6.6 kJ/mol), JUN (−6.4 kJ/mol), RELA (−7.7 kJ/mol), TP53 (−6.3 kJ/mol), BECN1 (−7.2 kJ/mol), SQSTM1 (−6.8 kJ/mol), MAP1LC3A (−7.1 kJ/mol), and MAP1LC3B (−6.2 kJ/mol; Figure 3E).

### 3.4. Effects of Fluoride on Homeostasis of Elements and MLT Intervention

**Figure 4:**
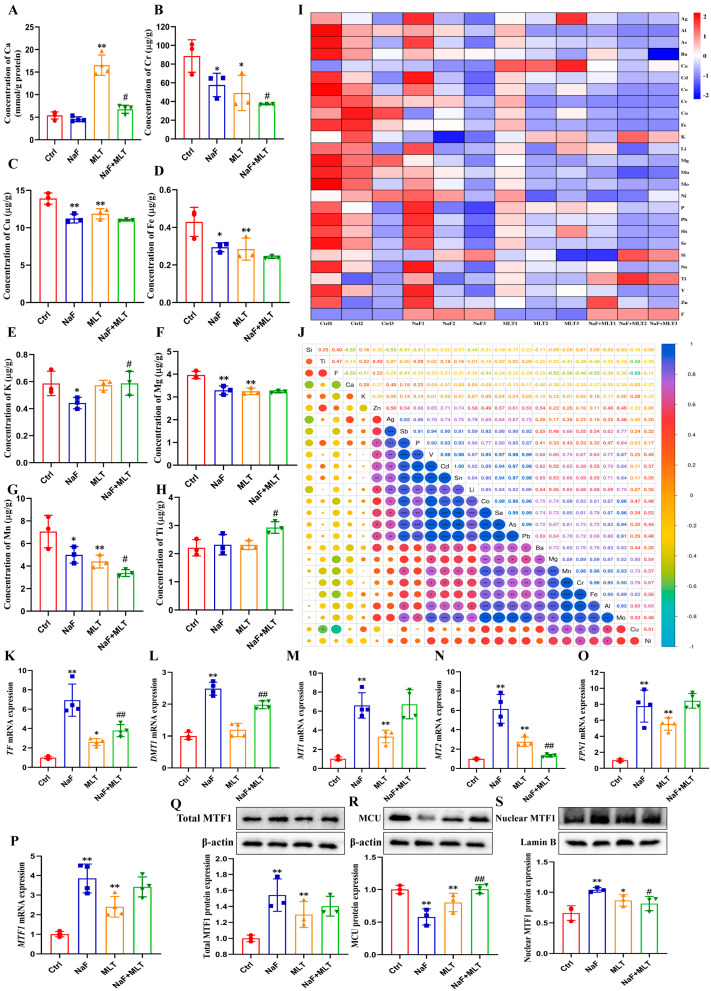
Fluoride induces ion homeostasis disorder and melatonin intervention in chickens. (**A**–**H**) Concentrations of calcium, chromium, copper, iron, potassium, magnesium, manganese, titanium in tibia of chicken. (**I**) Heat map of the concentrations of 26 elements in tibia of chicken. (**J**) Correlation analysis on the concentrations of 26 elements in tibia of chicken. (**K**–**P**) TF, DMT1, MT1, MT2, FPN1 and MTF1 mRNA expressions in tibia of chicken. (**Q**–**S**) Total MTF1, MCU, and nuclear MTF1 protein expressions in tibia of chicken. * *p* < 0.05, ** *p* < 0.01 and *** *p* < 0.001 indicate significant differences compared to the Ctrl group. ## *p* < 0.05 and # *p* < 0.01 indicate significant differences compared to the NaF group.

As shown in Figure 4A–I, element analysis data showed that fluoride treatment significantly reduced concentrations of Cr, Cu, Fe, K, Mg, and Mn (*p* < 0.05; *p* < 0.01). Ca concentration was significantly increased (*p* < 0.05), and Cr, Cu, Fe, Mg, and Mn concentrations were significantly decreased (*p* < 0.05; *p* < 0.01) following fluoride exposure. Whereas MLT administration significantly increased Ca concentration (*p* < 0.01), it significantly decreased the concentrations of Cr and Mg (*p* < 0.05; *p* < 0.01) in the fluoride-exposed group. Correlation analysis showed an obvious negative correlation between fluoride and Cu (*p* < 0.05; Figure 4J). After fluoride exposure, the mRNA expressions of TF, DMT1, MT1, MT2, FPN1, and MTF1, and protein levels of total and nuclear MTF1 were significantly increased (*p* < 0.01), whereas MCU protein expression was reduced, consistent with MLT supplement treatment (*p* < 0.05; *p* < 0.01; Figure 4K–S). MTL intervention significantly restored the effect of fluoride on TF, DMT1, MT2, MCU and nuclear MTF1 expressions (*p* < 0.05; *p* < 0.01; Figure 4K–S).

### 3.5. Fluoride-Induced Mitochondrial Damage and MLT Intervention

**Figure 5:**
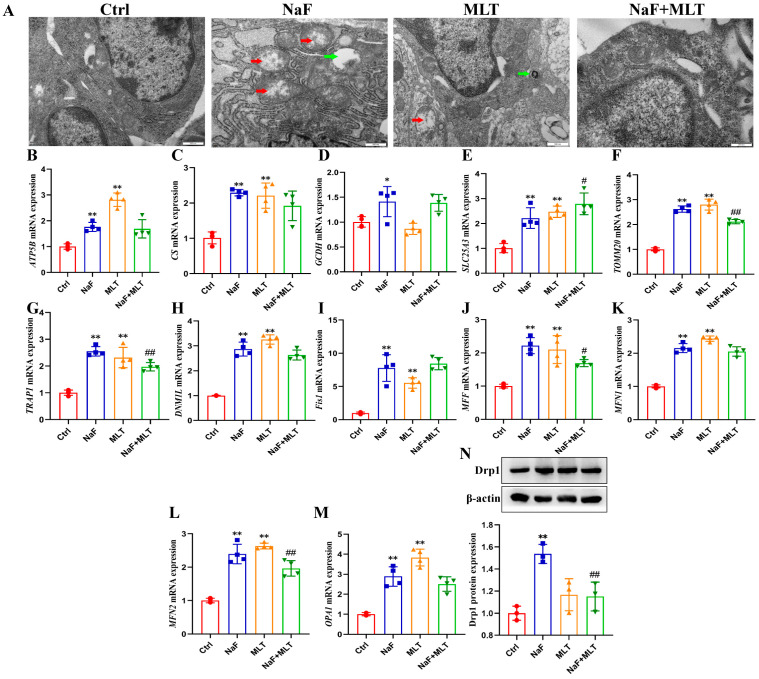
Fluoride induces mitochondrial damage and melatonin intervention in chickens. (**A**) The ultrastructure of the metaphysis of the chicken’s tibia, scale bars = 500 nm. The red arrows indicate mitochondrial ridge disappearance. The green arrows indicate autophagy. (**B**–**M**) ATP5B, CS, GCDH, SLC25A3, TOMM20, TRAP1, DNM1L, Fis1, MFF, MFN1, MFN2 and OPA1 mRNA expressions in tibia of chicken. (**N**) Drp1 protein expression in tibia of chicken. * *p* < 0.05 and ** *p* < 0.01 indicate significant differences compared to the Ctrl group. ## *p* < 0.05 and # *p* < 0.01 indicate significant differences compared to the NaF group.

As shown in Figure 5A, the mitochondria were normal in size and structurally intact in osteoblasts of the Ctrl, MLT, and NaF+MLT groups. Mitochondrial swelling, mitochondrial ridge disappearance (red arrow), and autophagy (green arrow) appeared in osteoblasts of NaF + MLT groups. Mitochondrial swelled, and mitochondrial ridge loss (red arrow) and autophagy (green arrow) occurred in NaF and MLT groups. After fluoride exposure, the mRNA expressions of ATP5B, CS, GCDH, SLC25A3, TOMM20, TRAP1, DNM1L, Fis1, MFF, MFN1, and MFN2, and Drp1 protein expressions were significantly increased (*p* < 0.05; *p* < 0.01), consistent with the effect of MLT (*p* < 0.01; Figure 5B–N). MTL intervention significantly restored the effect of fluoride on SLC25A3, TOMM20, TRAP1, MFF, MFN2, and Drp1 expressions (*p* < 0.05; *p* < 0.01; Figure 5B–N).

### 3.6. MLT Alleviated Fluoride-Induced Mitochondrial Autophagy

**Figure 6:**
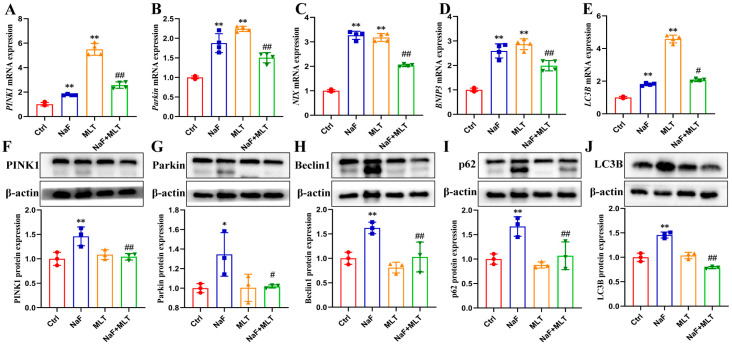
Fluoride induces mitophagy and melatonin intervention in chickens. (**A**–**E**) PINK1, Parkin, NIX, BNIP3, and LC3B mRNA expressions in tibia of chicken. (**F**–**J**) PINK1, Parkin, Beclin1, p62, and LC3B protein expressions in tibia of chicken. * *p* < 0.05 and ** *p* < 0.01 indicate significant differences compared to the Ctrl group. ## *p* < 0.05 and # *p* < 0.01 indicate significant differences compared to the NaF group.

After fluoride exposure, the mRNA expressions of PINK1, Parkin, NIX, BNIP3, and LC3B, were significantly increased (*p* < 0.01), consistent with the effect of MLT (*p* < 0.01). And the protein expressions of PINK1, Parkin, Beclin1, p62, and LC3B were higher (*p* < 0.05; *p* < 0.01; Figure 6) in the NaF group than those in the Ctrl group. MTL intervention significantly restored the effect of fluoride on PINK1, Parkin, NIX, BNIP3, and LC3B mRNA expressions, and PINK1, Parkin, Beclin1, p62, and LC3B protein levels (*p* < 0.05; *p* < 0.01; Figure 6).

### 3.7. Fluoride Caused Lysosome Injury and MLT Intervention

**Figure 7:**
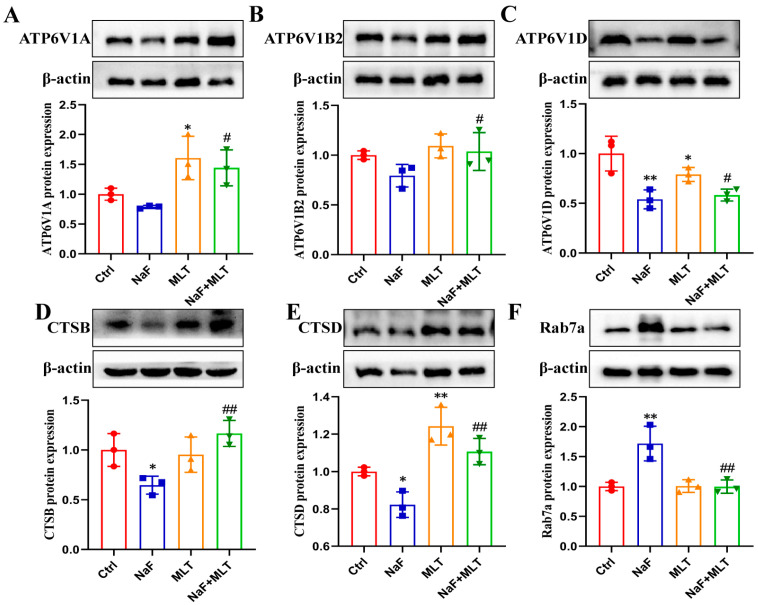
Fluoride induces lysosome injury and melatonin intervention in chickens. (**A**–**F**) ATP6V1A, ATP6V1B2, ATP6V1D, CTSB, CTSD, and Rab7a protein expressions in tibia of chicken. * *p* < 0.05 and ** *p* < 0.01 indicate significant differences compared to the Ctrl group. ## *p* < 0.05 and # *p* < 0.01 indicate significant differences compared to the NaF group.

Fluoride significantly decreased the protein expressions of ATP6V1D, CTSB, and CTSD (*p* < 0.05; *p* < 0.01; Figure 7A,D,E), and were significantly increased Rab7a protein level (*p* < 0.01; Figure 7F). MLT treatment significantly promoted ATP6V1A and CTSD protein expressions (*p* < 0.0; Figure 7A,E), and significantly reduced ATP6V1D level (*p* < 0.05; Figure 7C). MTL intervention significantly restored the effect of fluoride on ATP6V1A, ATP6V1B2, ATP6V1D, CTSB, CTSD, and Rab7a protein expressions (*p* < 0.05; *p* < 0.01; Figure 7).

## 4. Discussion

Fluoride demonstrates a well-established biphasic dose–response relationship in biological systems. At low doses concentrations, it helps prevent dental caries. This beneficial effect occurs through three main mechanisms: inhibiting tooth demineralization, suppressing plaque bacterial activity, and promoting enamel remineralization [32]. In contrast, long-term excessive fluoride exposure induces multisystem toxicity via intricate mechanisms. This toxicity poses serious threats to both human health and livestock productivity [33]. Previous research has demonstrated that fluoride accumulates in murine bones and induces bone injury through PINK1/Parkin-mediated mitophagy [14]. Nevertheless, the precise molecular mechanisms of fluoride-induced mitophagy in bone, and particularly melatonin’s (MLT) potential regulatory role, remain poorly understood. To address these knowledge gaps, a chicken model of fluoride exposure and/or MLT intervention was established. This study was carried out to explore MLT’s protective effects against fluoride-induced skeletal fluorosis via mitophagy modulation. Network pharmacology analysis identified mitophagy as a core pathway mediating MLT’s antagonistic effects on NaF-induced osteosclerosis. Our findings reveal that fluoride disrupts ion metabolism in chicken tibiae. This disruption subsequently activates PINK1/Parkin-regulated mitophagy. These findings provide novel insights into how fluoride causes bone damage and how MLT may offer therapeutic protection.

Chickens provide a robust translational model for fluoride toxicity research. The cellular mechanisms of fluoride-induced osteopathology and MLT’s osteoprotective effects are highly conserved across mammalian species, including humans. Therefore, findings from this chicken model offer critical implications for understanding skeletal fluorosis pathogenesis in higher vertebrates. They also help validate MLT’s therapeutic potential against fluoride-induced bone damage. Notably, fluoride bioaccumulation in poultry bones presents food safety implications. During thermal processing method like prolonged stewing, fluoride can leach from bone into edible matrices. This secondary contamination pathway may significantly increase dietary fluoride exposure in human populations. These findings highlight the need for thorough risk assessment and appropriate regulatory measures.

Bone is the primary site of fluoride accumulation in organisms. Therefore, bone fluoride concentration serves as a critical indicator for validating the establishment of a fluorosis model [34]. In the present study, NaF treatment significantly increased fluoride levels in chicken tibiae. This result confirms the successful creation of our chicken fluorosis model. Concomitantly, NaF exposure markedly enlarged the left tibial diameter. Histomorphological analysis further revealed irregular trabecular morphology, uneven trabecular thickness, and disorganized trabecular orientation in fluoride-exposed bones. These structural changes indicate enhanced but disorganized osteogenic activity, reflecting disrupted tibial growth and development. The pineal gland shows a particularly high affinity for fluoride accumulation. This organ’s high vascularization and position outside the blood–brain barrier allow it to reach the highest fluoride saturation levels among all organs [23]. This accumulation subsequently impairs MLT synthesis. Therefore, targeting fluoride-induced MLT deficiency represents a promising new strategy for preventing and managing skeletal fluorosis. In the current study, MLT intervention significantly reduced both left tibial length and body weight in NaF-exposed chickens. These findings align with previous research showing that MLT promotes lipolysis and reduces intramuscular fat deposition [35]. The weight reduction induced by MLT may thus be connected to its attenuation of osteogenic formation in the tibiae of fluorotic chickens. Collectively, these results demonstrate MLT’s dual role: it mitigates fluoride-induced bone abnormalities while also regulating body composition. These insights provide valuable guidance for developing nutritional interventions against fluorosis and for improving poultry product quality.

Upon absorption into the body, fluoride predominantly accumulates in hard tissues, especially bone, where it causes structural damage. The histopathological observations of tibial epiphyses confirmed this phenomenon in the present study. Importantly, MLT intervention substantially ameliorating fluoride-induced bone lesions. Fluoride impairs tibial load-bearing capacity by increasing bone density, which ultimately compromises bone mechanical strength [36]. Our results align with this mechanism. NaF treatment significantly increased BV/TV, Tb.N, and Conn.Dn. Simultaneously, it reduced Tb.Sp. MLT supplementation effectively counteracted all these NaF-induced changes [37]. Bone mechanical properties depend on its complex hierarchical structure. This structure includes cellular networks, mineralized fibrils, non-collagenous proteins, and water [38]. In our study, MLT significantly attenuated the NaF-induced increase in collagen volume fraction. This finding supports previous evidence that fluoride disrupts bone tissue homeostasis by promoting excessive collagen-dominated organic matrix deposition [39]. Together, these results confirm MLT’s protective role against fluoride-mediated bone damage. They highlight MLT’s potential as a nutritional intervention strategy to combat fluoride-induced bone abnormalities. This approach could improve poultry bone health and reduce food safety risks associated with fluoride bioaccumulation in animal-derived products.

Bone is a dynamically adaptive tissue maintained through continuous remodeling. This essential process balances bone resorption with new bone formation. Its regulation depends on the functional equilibrium among three key cell types: osteoblasts (bone-forming cells), osteoclasts (bone-resorbing cells), and osteocytes (mechanosensory cells) [40]. Osteoblasts are uniquely responsible for synthesizing bone matrix. Their differentiation is stringently controlled by the transcription factor Runx2 [41]. Runx2 drives mesenchymal stem cells to become mature osteoblasts by activating bone-specific matrix protein genes. These include genes encoding ALP, Col1a, and OCN [42]. Among these markers, ALP activity remains the most widely accepted indicator of osteoblast function. Meanwhile OCN (also known as bone gamma-carboxyglutamate protein, BGLAP) serves as a canonical bone formation marker. This protein is exclusively expressed by osteoblasts [43]. In the present study, MLT intervention significantly counteracted the effects of NaF exposure. It specifically suppressed the NaF-induced upregulation of OCN concentration, ALP activity and expression, BGLAP mRNA levels, and the protein expressions of Runx2 and Col1a. Together, these results demonstrate that MLT supplementation effectively mitigates fluoride-induced overactivation of osteoblast differentiation and abnormal bone formation. This evidence supports MLT’s protective role against fluoride-mediated bone pathology.

Trace and macroelements interact with bone nutrients to maintain normal bone metabolism. These elements are indispensable for proper bone growth and development [44]. Ca provides essential mineralization for bone structural integrity. It also acts as a reservoir to maintain stable systemic blood Ca levels. Fe contributes to bone matrix synthesis and the activation of 25-hydroxyvitamin D. Cu plays a critical in cross-linking collagen and elastin, which are key bone matrix components that determine bone strength and plastic deformation capacity. Mn serves as a cofactor for enzymes involved in cartilage ossification and mucopolysaccharide synthesis. Mg supports multiple functions in bone and tooth, maintain nerve-muscle homeostasis, promotes osteoblast proliferation and differentiation, and activates metabolic enzymes (including those regulating Ca and K metabolism). K itself helps prevent bone Ca loss, thereby enhancing bone integrity. Several elements serve additional roles in bone health. Cu, Zn, and molybdenum function as enzyme cofactors, while Cu, Zn, and Fe are key components of antioxidant enzymes linked to degenerative joint disease [45]. For instance, higher concentrations of Cu, Sr, and Zn have been detected in the femoral heads of severe osteoarthritis cases compared to mild cases [46]. Notably, the effects of NaF exposure and MLT intervention on macroelement, trace element, and toxic element concentrations in chicken tibiae have not been previously investigated. In the present study, NaF treatment significantly reduced tibial concentrations of Cr, Cu, Fe, K, Mg, and Mn. In contrast, MLT intervention counteracted these NaF-induced declines. It notably attenuated the reductions. It notably attenuated reductions in K, Ca, and Ti concentrations, and significantly reversed the decrease in Cr levels. These findings suggest that fluoride disrupts tibial ion homeostasis, which may partially explain its bone toxicity. MLT appears to alleviate these detrimental effects. Correlation analysis further revealed a strong negative association between tibial fluoride and Cu concentrations. This indicates an interaction between Cu and fluoride in chicken tibiae under both physiological and pathological conditions. Whether this negative correlation enables Cu to antagonize fluoride accumulation-related toxicity requires further investigation. Such research could provide novel insights into nutritional strategies for mitigating fluorosis.

MTF1 is a key regulatory protein. It controls the expression of metal transporters for elements like Fe and Cu. It also induces the transcription of metallothioneins (MTs), a family of stress-responsive proteins [23,47]. MTs play well-established roles in heavy metal detoxification and antioxidant defense. Previous studies suggest that MTs help maintain Fe and Cu homeostasis, providing protection against metal-induced toxicity and oxidative damage [48]. However, the roles of MTF1 and MTs in fluoride toxicity, and in MLT intervention, remains unknown. In the present study, MLT intervention significantly reduced the NaF-induced upregulation of MT1 and MT2 mRNA expression. It also attenuated the increase in nuclear MTF1 protein levels in chicken tibiae. These results indicate that fluoride exposure activates MTF1, which in turn induces MTs expression. The regulatory cascade is effectively suppressed by MLT supplementation. Beyond regulating MTs, MTF1 maintains systemic Fe homeostasis. It modulates the expression of transferrin (TF), a Fe-transporting glycoprotein, and ferroportin 1 (FPN1), the only known cellular Fe exporter [49]. Dysregulated Fe metabolism is associated with an increased risk of bone disorders [50]. Moreover, divalent metal transporter 1 (DMT1) regulates Fe homeostasis in osteoblasts, ultimately influencing their function [51]. Normal cellular Fe homeostasis depends on the equilibrium Fe import and export. This balance prevents both Fe overload and deficiency. Functionally, FPN1 mediates Fe efflux from cells into the circulation, while TF delivers Fe to target tissues and cells [52]. A previous study in fish livers reported a strong positive correlation between MTF1 and TF expression, suggesting their coordinated role in Fe transport [53]. Consistent with these findings, our results showed that NaF treatment significantly upregulated TF and FPN1 gene expression, yet reduced tibial Fe and Cu concentrations. MLT partially reversed these effects, specifically normalizing the elevated TF levels. Together, these data suggest that fluoride exposure disrupts metal homeostasis by perturbing MTF1-mediated metal response pathways, contributing to bone damage. In contrast, MLT alleviates fluoride-induced bone toxicity by inhibiting MTF1-dependent metal response cascade and restoring Fe and Cu homeostasis.

Ca homeostasis is fundamental for maintaining normal skeletal function and integrity. Prolonged fluoride exposure disrupts this balance in bones and teeth [14]. Previous research shows that dietary Ca supplementation (1%) alleviates fluoride-induced bone damage in rats. This protective effect occurs through modulation of endoplasmic reticulum stress and the PI3K/AKT pathway [54,55]. In the present study, MLT intervention significantly elevated tibial Ca concentrations in NaF-exposed chickens. This suggests that MLT mitigates NaF-induced bone damage, at least partially, by restoring Ca homeostasis. Mitochondria play a central role in maintaining intracellular Ca homeostasis. The mitochondrial calcium uniporter (MCU) transports cytoplasmic Ca into mitochondria. This Ca flux regulates multiple cellular processes, including energy metabolism, ROS generation, mitochondrial dynamics, and cell survival/death decisions [56]. Key biomarkers of mitochondrial function include: ATP synthase subunit β (ATP5B, involved in energy metabolism [57]), citrate synthase (CS, rate-limiting enzyme of the tricarboxylic acid cycle regulating mitochondrial respiration [58]), tumor necrosis factor receptor-associated protein 1 (TRAP1, a chaperone maintaining mitochondrial metabolic homeostasis [59]), solute carrier family 25 member 3 (SLC25A3, mediating phosphate transport for ATP synthesis [60]), glutaryl-CoA dehydrogenase (GCDH, regulating amino acid metabolism [61]), and translocase of the outer mitochondrial membrane 20 (TOMM20, involved in mitochondrial protein import [62]). Mitochondrial dynamics (fusion/fission) are critical for preserving mitochondrial function under metabolic or environmental stress [63]. Fusion is regulated by mitofusin-1 (MFN1), mitofusin-2 (MFN2), and optic atrophy protein 1 (OPA1), while fission is governed by mitochondrial fission 1 (FIS1), dynamin-related protein 1 (Drp1), and mitochondrial fission factor (MFF) [64]. Sustained cytoplasmic Ca elevation activates calcineurin-mediated dephosphorylation of Drp1 (S637 residue), promoting Drp1 translocation to mitochondria and inducing fission [65]. In this study, NaF treatment significantly downregulated MCU protein expression and upregulated the levels of ATP5B, CS, GCDH, SLC25A3, TOMM20, TRAP1, DNM1L (Drp1 encoding gene), FIS1, MFF, and Drp1—effects reversed by MLT. Notably, NaF exposure also increased MFN1, MFN2, and OPA1 expression, while MLT reduced NaF-induced MFN2 upregulation. These results indicate that NaF induces mitochondrial fragmentation via promoting fission, leading to mitochondrial dynamic imbalance. MLT counteracts this by regulating Ca influx and mitochondrial function. The NaF-induced upregulation of fusion-related proteins (MFN1/2, OPA1) likely reflects a negative feedback mechanism to maintain mitochondrial homeostasis, with MLT’s role in restoring calcium homeostasis and mitochondrial dynamics to alleviate fluoride-induced bone toxicity. This insight contributes to developing nutritional strategies for improving poultry bone health and food safety.

Mitochondrial dysfunction drivers of bone aging and pathology. This dysfunction includes metabolic impairment, structural damage, and defective quality control [66]. Mitochondrial fission serves as a key quality control mechanism. It separates damaged mitochondrial segments from healthy regions. These damaged mitochondrial segments are then eliminated via mitophagy, preserving overall mitochondrial homeostasis [67]. This process involves autophagosomes engulfing damaged mitochondria. The autophagosomes then fuse with lysosomes to complete degradation [68]. In recent years, mitophagy has become the focus of research on understanding bone diseases [69]. Multiple pathways regulate mitophagy, with the ubiquitin-dependent PINK1/Parkin pathway being the most well-characterized [69]. This pathway mediates autophagosome-lysosome fusion, and initiates mitophagy through interactions with Rab7A-specific guanine nucleotide exchange factors [70]. Lysosomal function is equally critical for efficient mitophagy. Cysteine proteases like cathepsin B (CTSB) and cathepsin D (CTSD) degrade unwanted proteins [71]. Meanwhile, lysosomal acidification maintains protease activity. Proteins such as ATP6V1A (ATPase, H+ transporting, lysosomal V1 subunit A), ATP6V1B2 (ATPase, H+ transporting, lysosomal V1 subunit B, isoform 2), and ATP6V1D (ATPase, H+ transporting, lysosomal V1 subunit D) sustain this acidification [72]. Notably, mitophagy dysfunction worsens lysosomal acidification failure, creating a vicious cycle of impaired organelle quality control [73]. In the present study, transmission electron microscopy (TEM) observations revealed intact mitochondrial morphology and normal size in the control (Ctrl) and NaF+MLT groups. In contrast, the NaF group exhibited prominent mitochondrial swelling, cristae loss (red arrow), and autophagic structures (green arrow). Supporting these findings, network pharmacology analysis identified mitophagy as a core pathway mediating MLT’s protective effects against NaF-induced osteosclerosis. Molecular analysis showed that NaF treatment significantly upregulated the expression of mitophagy-related proteins (PINK1, Parkin, NIX, BNIP3, Beclin1, p62, LC3B, Rab7A), while downregulating lysosomal markers (ATP6V1D, CTSB, CTSD). MLT intervention completely reversed these changes. These results demonstrate that MLT alleviates fluoride-induced bone damage by inhibiting PINK1/Parkin-mediated mitophagy and restoring mitochondrial–lysosomal function. This aligns with previous findings in mammalian models [14]. Our findings support MLT’s potential as a nutritional intervention against fluoride-mediated bone toxicity in poultry. This approach could benefit both animal health and the safety of poultry-derived food products. Moreover, using a chicken model with high-dose drug intervention as the research subject is necessary to clarify the specific toxicological characteristics of NaF and the protective mechanism of MLT. However, caution must be exercised when directly extrapolating these findings to human health risks associated with low-level fluoride exposure. Future studies employing environmentally relevant, low-dose chronic exposure paradigms are warranted to confirm the translational relevance of the mechanisms identified here.

A pivotal and unexpected finding of this study was that MLT monotherapy, at the dose utilized for protection (25 mg/kg/day), functioned as a potent metabolic stimulator, inducing comprehensive remodeling of healthy bone. Contrary to the paradigm of MLT as a universally benign agent, its administration alone elicited significant changes across a wide spectrum of parameters. It significantly increased bone morphology indices (tibial diameter, collagen volume fraction) and concentrations of OCN and Ca, while paradoxically also elevating bone fluoride levels. This was accompanied by a systemic reprogramming of cellular metabolism, evidenced by the upregulation of bone formation-related genes (BGLAP, Runx2, Col1a), altered trace element homeostasis (decreased Cr, Cu, Fe, Mg, Mn), and the widespread activation of the mitochondrial quality control system—including markers of function (ATP5B, CS), dynamics (DNM1L, MFN1/2, OPA1), and PINK1/Parkin-mediated mitophagy. The presence of autophagic structures, confirmed by TEM, provided ultrastructural support for this activation. This is a pivotal observation, suggesting that MLT’s role in bone metabolism may be biphasic. At this dosage, MLT may act as a mild stressor, potentially activating adaptive pathways such as mitophagy in the absence of an overt toxicant. Our data thereby move beyond a simplistic protective–toxic dichotomy and highlight the critical importance of defining a therapeutic window for MLT. The effects seen here underscore that ‘more is not always better,’ and the dosage is a paramount factor determining the outcome of MLT supplementation. This profound biological impact is not a mere inconsistency but a critical result that suggests a biphasic or hormetic role for MLT in bone metabolism. At this high dosage, MLT appears to act as a mild stressor, potentially activating adaptive pathways in the absence of an overt toxicant. Our data thereby move beyond a simplistic protective–toxic dichotomy and fundamentally reframe the significant effects of MLT monotherapy as both a key mechanistic insight and a critical limitation of the current therapeutic approach. The use of a single, high dose precludes conclusions about safety and underscores that ‘more is not always better’. Therefore, defining the precise safe and effective therapeutic window for MLT is an absolute prerequisite for any potential practical application. This highlights an urgent need to clarify the dosage range and treatment duration for MLT, particularly as a nutritional intervention in poultry. Consequently, the central imperative for future research, directly highlighted by our findings, must be systematic dose–response studies. Such work is essential to delineate the precise dosage at which MLT’s protective effects against bone toxicants like NaF are maximized, while its potential to induce stress responses in healthy tissue is minimized. Establishing this window is a crucial step for ensuring animal health and food safety, and for any potential translation of MLT as a bone-protective agent.

## 5. Conclusions

**Figure 8:**
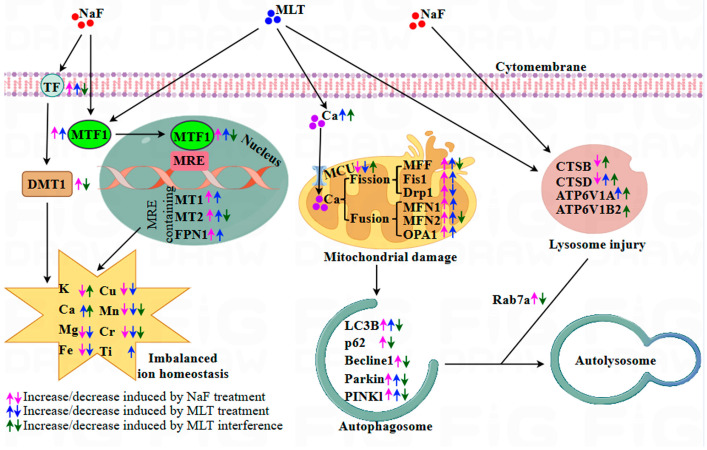
Schematic diagram of the proposed mechanism of melatonin to alleviate fluorine-induced bone toxicity via ion homeostasis imbalance and PINK1/Parkin-mediated mitophagy.

This study explored how MLT protects against NaF-induced skeletal fluorosis in chickens. It also evaluated the long-term effects of MLT supplementation alone. NaF exposure successfully induced skeletal fluorosis in chicken. This was confirmed by several key observations: elevated fluoride levels in tibiae, disrupted bone microarchitecture, abnormal osteoblast activity, imbalanced ion levels, impaired mitochondrial function, and activated PINK1/Parkin-mediated mitophagy. MLT treatment effectively counteracted these NaF-induced detrimental effects. It worked by restoring ion homeostasis, preserving mitochondrial dynamics, and inhibiting excessive mitophagy. Network pharmacology confirming mitophagy as a key pathway for MLT’s protection (Figure 8). Notably, long-term high-dose MLT alone disrupted tibial trace elements, promoted abnormal bone formation, and activated mitophagy in healthy chickens, highlighting the need to define MLT’s safe dosage and treatment duration for poultry. This work offers a nutrition-based strategy (MLT) to reduce fluoride-related risks in poultry bones and improve poultry health, supporting practices that protect both poultry productivity and human dietary safety.

## Data Availability

The original contributions presented in the study are included in the article/Supplementary Materials, further inquiries can be directed to the corresponding authors.

## Supplementary Materials

The following supporting information can be downloaded at: https://www.mdpi.com/article/10.3390/foods14244173/s1, Table S1: Concentration of fluoride in the formulated feeds; Table S2: Concentration of nutrient composition in the formulated feeds; Table S3: Primer sequences for qRT-PCR; Table S4: Network Pharmacology GO Enrichment Results; Table S5: Network Pharmacology KEGG Enrichment Results; Figure S1: GO enrichment network map of MLT on NaF-induced osteosclerosis target genes.
