# Supplementary material for: Melatonin Supplementation Relieves Fluoride-Induced Bone Injury via Ion Homeostasis Disorder and PINK1/Parkin-Mediated Mitophagy
Source: Foods. 2025 Dec 5;14(24):4173. doi: 10.3390/foods14244173 (PMC12731749; doi:10.3390/foods14244173)
Supplement: Supplementary file 1 [file foods-14-04173-s001.zip › foods-3969838-supplementary.pdf]

Supplementary Materials

Melatonin Supplementation Relieves Fluoride-Induced Bone Injury via Ion Homeostasis Disorder and PINK1/Parkin-Mediated Mitophagy

Table S1 Concentration of fluoride in the formulated feeds

Production	Concentration of fluoride
211 (0-6 weeks)	18.13 ± 0.86
212 (7-15 weeks)	8.35 ± 1.20
218 (16 weeks)	8.90 ± 1.11

Table S2 Concentration of nutrient composition in the formulated feeds

Ingredients	Product name		
	211 (0-6 weeks)	212 (7-15 weeks)	218 (16 weeks)
Crude protein (% ≥)	19.5	15.0	16.5
Coarse fibre (% ≤)	5.5	8.0	5.0
Crude ash (% ≤)	8.0	10.0	15.0
Calcium (%)	0.8-1.2	0.8-1.4	3.0-4.5
Phosphorus without phytate phosphorus (% ≥)	0.5	0.5	0.5
Phosphorus without phytate phosphorus (% ≥)	0.3	0.3	0.3
Sodium chloride (%)	0.3-0.8	0.3-0.8	0.3-0.8
Methionine (%)	0.45-0.90	0.30-0.90	0.38-0.90

Table S3 Primer sequences for qRT-PCR

Gene	Primer sequences (5’>3’)	Product size (bp)	Accession No.
GAPDH	F: CAGAACATCATCCCAGCGTCCAC<br>R: CGGCAGGTCAGGTCAACAACAG	134	NM_204305.2
RUNX2	F: GCAACAGCAACAGCAGCAAGAAG<br>R: CAGCACGGAGCACAGGAAGTTG	136	NM_204128.2
BGLAP	F: AGGCAGAAGCGGCACTACG<br>R: GGCTCAGCTCACACACCTCTC	91	NM_205387.4

Col1a	F: GCGACAGCGGCAACATCC<br>R: GACCCTCTACTCCAGCACTCTC	150	NM_001396622.1
ALP	F: CGTCAGCCGTGTGGACTTCC<br>R: GGTCGGTCTCGTTGTTCCTGTC	83	NM_205360.2
DNM1L	F: ATGAGACCTTTGGACGAACC<br>R: AACAAACAAGGCAGGACGG	113	NM_001079722.2
MFF	F: GTCTTGGATGTGCTGGATGA<br>R: TGAGGGTTAGAGGAAGTGGTAG	77	XM_040679322.2
FIS1	F: GGAGTTTGACGATGTGGTGG<br>R: CGCACGTCGCTGTTGTATCT	147	XM_040657193.2
MFN1	F: AGCGTCAAGACCGTCAATC<br>R: CTGTCCACTAAGACCAAATCGT	134	XM_046923917.1
MFN2	F: CAGTGGCATTAGTGAAGTGTTG<br>R: GGACTTTGTCCCATAGCATG	110	XM_040689233.2
OPA1	F: CCAGGGTTATGACAGCGACA<br>R: GCATCCGTTGGTATTTTAGC	107	NM_001039309.2
LC3B	F: TTACACCCATATCAGATTCTTG<br>R: ATTCCAACCTGTCCCTCA	143	XM_040688401.2
PINK1	F: TGCAGTTGTTGGAAGGTGTG<br>R: CAGCCAGCAGAATCGAACTC	100	NM_001389481.2
Parkin	F: GTCCAGCAAAGCATCGTTCA<br>R: CAACGATGGAAGGATGCTGG	159	XM_046914604.1
NIX	F: TGGGTGGAGTTGCAGATGA<br>R: GAATGGAGGATGATGATGGG	97	NM_001030885.3
BNIP3	F: TTTTCAAACACCCCAGACG<br>R: TGTAAATCCCGAGTCCAATG	150	XM_040674209.2
ATP5B	F: GTCTACGGACAGATGAATGAGC<br>R: CTGGGTGAAGCGGAAAAT	138	NM_001031391.3
CS	F: TACTACACGGTGCTCTTCGG<br>R: GCAGTGCTCATGGATTTGG	110	XM_040693724.2
TOMM20	F: CCCTATTCATCGGCTACTGC<br>R: TGTTTCTTCCTTCGCTCCC	91	XM_040668523.2
SLC25A3	F: AGGTGTCTGGAAAGGTCTGTT<br>R: GTGGACGAGGAAGTCTGAAATA	110	XM_046907363.1

GCDH	F: TACGACAAAGCCACCAAGAG<br>R: CAGCAAGAAACCCCGAAT	129	XM_040693082.2
TRAP1	F: GGATATTGTTGCCCGTTCC<br>R: TCTGCCATTAGCCGATGA	108	XM_046927481.1
TF	F: TACAAGCTGAAGCCCATTGC<br>R: TCTTGCCCTGCAAGTCGTT	124	NM_205304.2
MT1	F: GACTGCCCTTGTGCCACCG<br>R: TGCACACTTGGCACATCCT	126	NM_001097538.2
MT2	F: TGAACCATGGACCCTCAGGA<br>R: AGCCCTTGGCACAGTTGTT	151	NM_205275.2
MTF1	F: AAGGCGCAACACTCACTTTG<br>R: TCGTGGACAGCCCTCAAAAG	92	NM_001031495.2
FPN1	F: TTGTGGTTGCAGGGGAAGAC<br>R: GCACATCGACATCAGGTTCC	173	NM_001012913.2
DMT1	F: CTCATCACCATCGCCGACACC<br>R: AACGCCTCCAGCTTCCGCAGA	71	NM_001396394.1

Table S4 Network Pharmacology GO Enrichment Results

Go Name	GO ID	GO Category	pvalue
response to nutrient levels	GO:0031667	BP	2.75E-34
positive regulation of programmed cell death	GO:0043068	BP	2.00E-29
response to steroid hormone	GO:0048545	BP	1.10E-28
cellular response to cytokine stimulus	GO:0071345	BP	2.53E-28
regulation of apoptotic signaling pathway	GO:2001233	BP	3.98E-28
response to peptide hormone	GO:0043434	BP	2.96E-27
cellular response to abiotic stimulus	GO:0071214	BP	3.66E-27
cell population proliferation	GO:0008283	BP	7.94E-27
regulation of smooth muscle cell proliferation	GO:0048660	BP	6.18E-25
positive regulation of cell migration	GO:0030335	BP	6.34E-25

death-inducing signaling complex	GO:0031264	CC	1.97E-06
pseudopodium	GO:0031143	CC	1.87E-05
cyclin-dependent protein kinase holoenzyme complex	GO:0000307	CC	2.68E-05
platelet alpha granule lumen	GO:0031093	CC	4.44E-05
germ cell nucleus	GO:0043073	CC	4.70E-05
organelle outer membrane	GO:0031968	CC	4.11E-09
PML body	GO:0016605	CC	0.000303843
autophagosome	GO:0005776	CC	0.000384207
vesicle lumen	GO:0031983	CC	3.30E-09
endoplasmic reticulum lumen	GO:0005788	CC	2.83E-08
ubiquitin protein ligase binding	GO:0031625	MF	8.39E-22
protein domain specific binding	GO:0019904	MF	1.28E-16
transcription factor binding	GO:0008134	MF	4.32E-16
receptor ligand activity	GO:0048018	MF	2.85E-13
kinase regulator activity	GO:0019207	MF	2.41E-12
phosphatase binding	GO:0019902	MF	2.20E-10
protein homodimerization activity	GO:0042803	MF	3.13E-10
protease binding	GO:0002020	MF	6.39E-09
phosphotransferase activity, alcohol group as acceptor	GO:0016773	MF	7.36E-08
protein heterodimerization activity	GO:0046982	MF	1.24E-07

Table S5 Network Pharmacology KEGG Enrichment Results

KEGG Name	KEGG ID	Term PValue
Pathways in cancer	KEGG: 05200	0.018524
Amyotrophic lateral sclerosis	KEGG: 05014	0.008772
Lipid and atherosclerosis	KEGG: 05417	0.002552

Transcriptional misregulation in cancer	KEGG: 05202	0.002809
JAK-STAT signaling pathway	KEGG: 04630	0.036263
Necroptosis	KEGG: 04217	0.022153
Cell cycle	KEGG: 04110	0.008502
Fluid shear stress and atherosclerosis	KEGG: 05418	0.005972
Mitophagy - animal	KEGG: 04137	2.94E-04
Hypertrophic cardiomyopathy	KEGG: 05410	2.75E-04
Longevity regulating pathway	KEGG: 04211	0.006749
PPAR signaling pathway	KEGG: 03320	3.40E-06
Platinum drug resistance	KEGG: 01524	2.89E-05
Epithelial cell signaling in Helicobacter pylori infection	KEGG: 05120	0.001933
Chemical carcinogenesis - DNA adducts	KEGG: 05204	0.013853
Inflammatory bowel disease	KEGG: 05321	0.024247
Regulation of lipolysis in adipocytes	KEGG: 04923	0.024247
Type II diabetes mellitus	KEGG: 04930	0.027581
Thyroid cancer	KEGG: 05216	0.041784
Asthma	KEGG: 05310	0.003842

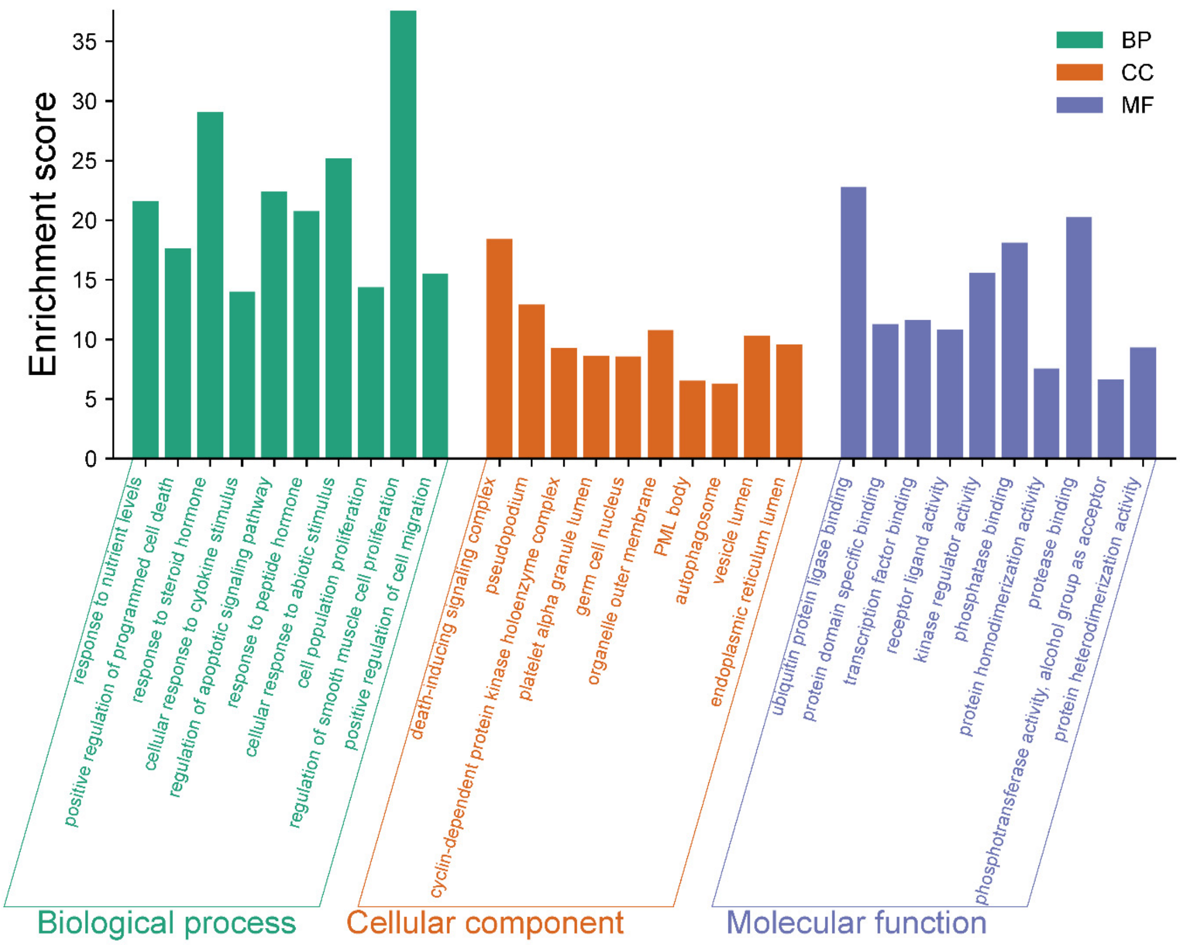

Figure S1 GO enrichment network map of MLT on NaF-induced osteosclerosis target genes.
